# Genomic Evidence for Direct Transmission of *mecC*-MRSA between a Horse and Its Veterinarian

**DOI:** 10.3390/antibiotics12020408

**Published:** 2023-02-17

**Authors:** Ervin Albert, Judit Sahin-Tóth, Andrea Horváth, Márton Papp, Imre Biksi, Orsolya Dobay

**Affiliations:** 1 Department of Pathology, University of Veterinary Medicine Budapest, Dóra Major, HU-2225 Üllő, Hungary; 2 Institute of Medical Microbiology, Semmelweis University, Nagyvárad Tér 4., HU-1089 Budapest, Hungary; 3 Centre for Bioinformatics, University of Veterinary Medicine Budapest, István u. 2., HU-1078 Budapest, Hungary

**Keywords:** *Staphylococcus aureus* carriage, MRSA carriage, *mecC*, whole-genome sequencing, horse, veterinarian

## Abstract

Methicillin-resistant *Staphylococcus aureus* bearing the *mecC* gene (*mecC*-MRSA) has been reported from animals and humans in recent years. This study describes the first *mecC*-MRSA isolates of human and equine origin in Hungary (two isolates from horses and one from a veterinarian, who treated one of the infected horses, but was asymptomatic). MRSA isolates were identified by cultivation and PCR detection of the species-specific *spa* gene and *mecA*/*mecC* methicillin resistance genes. The isolates were characterized by antibiotic susceptibility testing, MLST, *spa*, *SCCmec* typing, PFGE and whole genome sequencing (WGS). All three isolates belonged to the ST130-t843-*SCCmec* XI genotype, and carried the *mecC* and *blaZ* genes. Apart from beta-lactam drugs, they were sensitive to all tested antibiotics. The isolates of the infected horse and its veterinarian had the same PFGE pulsotype and showed only slight differences with WGS. Hence, this is the first description of direct transmission of a *mecC*-carrying MRSA between a horse and its veterinarian. The emergence of *mecC* in the country highlights the importance of the appropriate diagnostics in MRSA identification.

## 1. Introduction

*Staphylococcus aureus* is one of the most important pathogenic bacteria in humans, causing a wide range of suppurative infections, from milder skin and soft tissue inflammations to life threatening systemic diseases [1]. Although, in most animals, *S. aureus* infections are quite rare, in cattle it frequently causes bovine mastitis [2]. In other animal species such as horses and companion animals, the pathogen can be isolated from cutaneous, eye, urinary tract, joint and bone infections, pneumonia and surgical wound infections [3,4]. 

*S. aureus* is not only capable of causing severe infections, it is often also difficult to treat due to its resistance to various antibiotics. Methicillin-resistant *S. aureus* (MRSA)was first described in 1961, and since has become a global epidemiological threat, and can be responsible for nosocomial outbreaks as well [5,6].

The first cases of MRSA infection in horses were reported in the United States and in Japan in 1997 [7,8]. Nosocomial outbreaks in equine clinics were then documented in the United States in 1999, followed by Canadian and Central European cases a few years later [9,10,11]. In Europe, MRSA infections in horses remained associated mainly with veterinary healthcare settings [11,12,13]. 

Nasal MRSA colonization of veterinarians treating horses was reported as well [9,11,14]. In a Canadian veterinary hospital, even MRSA skin and soft tissue infections were detected among the hospital staff [12].

In MRSA, resistance to β-lactams is mediated by a modified penicillin binding protein, in most cases PBP2a, coded by the *mec*A gene [5]. However, the *mec*A gene is not the only one resulting in methicillin resistance in *S. aureus*. In 2007, a new *mecA* gene homologue, *mecA*_LGA251_, was found in *S. aureus* isolates from bulk cow milk samples in the UK and, in 2010, the same gene was detected in two human clinical isolates in Ireland [15,16]. This novel *mec* gene showed approximately 69% sequence identity to the original *mecA* gene sequences [5]. The new *mec* variant was named *mecC* in 2012 and the protein product of the gene is called PBP2c [17]. The *mecC* gene is located on a new *Staphylococcal Cassette Chromosome (SCC) mec* element, *SCCmec* type XI [15,16].

During a retrospective study of phenotypically MRSA isolates lacking the *mecA* gene, the *mecC* gene was discovered in a strain deriving from blood stream infection, originally isolated in 1975 in Denmark. This finding suggests that although *mecC*-MRSA was only identified recently, it has been present for over 40 years [15,16]. In recent years, *mecC*-MRSA isolates have been reported from several European countries from livestock (mainly cattle [15]), horses [18], wild animals such as hedgehogs [19,20], otters, brown hares [21], wild boars and fallow deer [22], as well as from environmental samples [5,22,23]). Although the majority of *mecC*-MRSA isolates were described in Europe, in the recent years it was reported from Africa, America, Asia, and Australia as well [24,25,26,27,28].

The *mecC* gene and *SCCmec* type XI are mainly associated with multilocus sequence typing (MLST) clonal complexes CC130 and CC425, both in humans and animals. However, *mecC*-MRSA belonging to other CCs has also been detected, including CC1943, CC599, CC97, CC59, and CC49 [5,15,22,26,29]. The most common *spa* (Staphylococcal protein A) type associated with *mecC*-positive strains is t843 [15,29]. The first *mecC*-MRSA infections in horses were reported from France and Germany and belonged to CC130 and CC49 [12,18]. CC130 is described to be an animal specific clonal complex; nevertheless, it has the capacity to spread to humans during zoonotic transmission, posing an occupational risk for infections of veterinarians and farmers [30,31]. In nosocomial *mecA*-MRSA cases, the strains were characterized by the MLST type ST8, with *spa* type t064 and *SCCmec* IV in Northern America, while European isolates belonged to ST254-t036 with *SCCmec* IV or to CC398-t011/t6867, or to ST8-t036 with *SCCmec* IV, and the *aacA-aphD* aminoglycoside resistance gene was frequently detected in them [11,12]. Other genotypes, such as CC1-t127, CC22, CC130 and CC225 were also identified in and outside the clinics, though with much lower frequencies [13].

The *mecC*-MRSA infection currently appears to be uncommon in humans, and its prevalence is reported to be 0–2.8% [29,32]. Animal contact is the major risk factor for *mecC*-MRSA infection or carriage in humans; thus, it can be regarded as a zoonotic or livestock associated pathogen [29]. In several human cases, the patients had known contact with *mecC*-MRSA carrying ruminants (dairy cattle and sheep), and they lived in an area with a high density of farms or were working as a veterinarian [33]

In this study we report *mecC*-MRSA strains obtained from horses and a veterinarian in Hungary. We provide whole-genome sequencing (WGS) data of the isolates with phylogenetic insight, assessing the relation of the isolates to each other and to other *mecC*-MRSA isolates. 

## 2. Results

### 2.1. Bacterial Samples and Clinical Background of the mecC-MRSA Isolates

**Figure 1:**
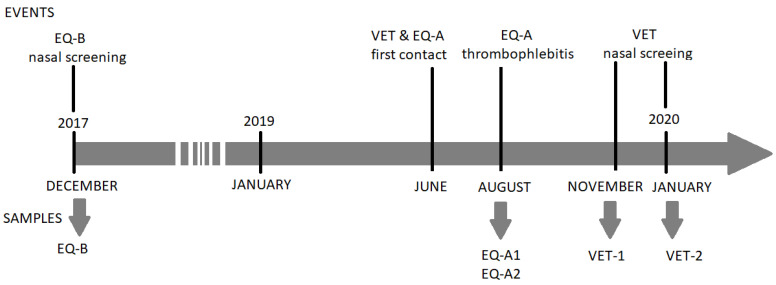
Timeline of the sampling procedures.

One of the horses (EQ-A) had a history of chronic obstructive pulmonary disease (COPD). This 19-year-old mare was a patient of a private veterinary practice located in western Hungary and was first visited by the veterinarian to apply intravenous hypertonic sodium chloride infusion therapy lasting for one week at the end of June 2019 (Figure 1). The infusion was administered in the left jugular vein through a long-term intravenous cannula on several consecutive occasions. Of note, the microbiological examination of bronchoalveolar and tracheal lavage samples taken shortly before the therapy returned no pathogenic bacteria. After the treatment, the horse was next visited by the veterinarian at the beginning of August 2019 because of thrombophlebitis of the formerly cannulated jugular vein. The microbiological testing, performed at a private veterinary microbiological diagnostic laboratory (DUO-BAKT Laboratory, Budapest, Hungary) revealed MRSA infection, yielding the EQ-A1 isolate. A control visit and microbiological sampling took place 21 days later and yielded the isolate EQ-A2 (Figure 1). According to the next visit in the beginning of October, the thrombophlebitis did not resolve, and the local circulation did not restore, however no further complication was observed. The veterinarian did not visit the horse between August and October.

The first nasal sample (VET-1) was taken from the veterinarian in November 2019, as part of a voluntary screening. It came to our attention only later that this equine veterinarian was the attending therapist of EQ-A in August 2019. Therefore, a control follow-up sample was requested from the veterinarian in January 2020 (VET-2), and the two isolates were analysed together (Figure 1). In the timeframe of June 2019–January 2020, the veterinarian did not take any antibiotic. However, a nasal decolonisation therapy using mupirocin ointment was applied after the control sampling, resulting in a third consecutive, yet negative bacteriological culture. 

The other horse (EQ-B), a 9-year-old gelding, was admitted to the Department and Clinic of Equine Medicine (DCEM, Üllő, Hungary) because of severe colic symptoms in December 2017 (Figure 1). The colic was diagnosed as inoperable, and the horse was subsequently euthanized. A nasal swab was taken from the animal as part of a bacteriological screening campaign of horses in the DCEM, shortly before the euthanasia. This horse had no co-morbidities linked to MRSA, and was thus regarded as an asymptomatic carrier. 

No epidemiological link could be discovered between the two horses, EQ-A and EQ-B.

### 2.2. Identification, Typing and Antimicrobial Susceptibility of the Isolates

**Figure 2:**
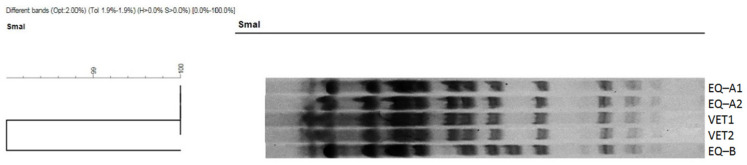
The PFGE dendogram of the *mecC*-MRSA isolates. All isolates, except EQ-B, clustered together and had the same banding pattern. For the original gel picture, please see Figure S1.

Isolates EQ-B, VET-1, and VET-2 grew on the selective chromogenic agar plates, though each needed more than 24 h to be visible on the plates and one more day to reach the characteristic medium size and pink to mauve colour. Of note, isolates EQ-A1 and EQ-A2 also had a longer culturing time on the Columbia blood agar at the time of their primary isolation (>24 h). According to the multiplex PCR, all isolates carried the *mecC* and the *spa* genes but lacked the *lukS-PV/lukF-PV* gene determinant. All isolates were negative for *mecA. Spa* typing resulted in t843 in all isolates, but PFGE revealed some differences: while all other isolates had the same banding pattern, EQ-B differed from them slightly (Figure 2). All *mecC*-MRSA were phenotypically resistant to beta-lactam antibiotics, except the amoxicillin-clavulanic acid combination (≤2/1 mg/L). They were also susceptible to the majority of the other antimicrobial agents tested. For further details, please refer to Table S1.

### 2.3. Genetic Relatedness, Resistance, and Virulence Genes of Whole Genome Sequenced Strains

**Figure 3:**
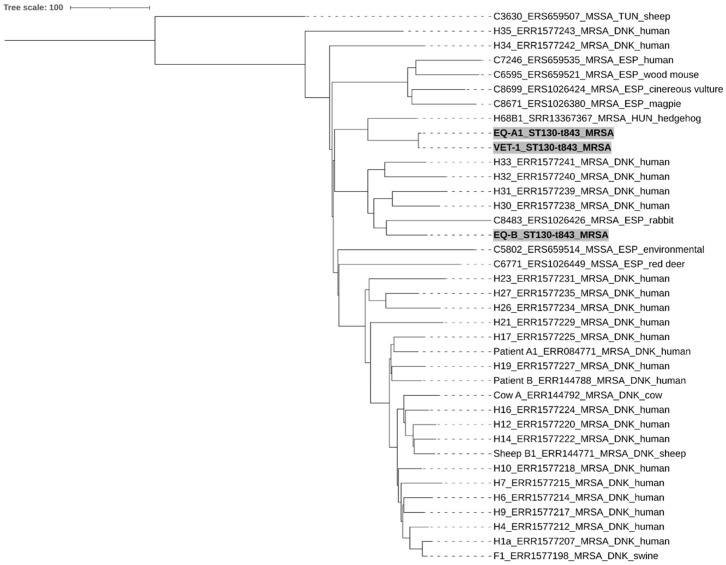
Neighbour-joining tree of CC130 *S. aureus* isolates. Genetic relationship between the two equine (EQ-A1, EQ-B) and the human (VET-1) originated from *mecC*-positive isolates from Hungary (highlighted with grey background), in comparison to other selected 32 *mecC*-positive MRSA and three MSSA CC130 isolates from Europe (Denmark (DNK), Hungary (HUN), Spain (ESP), and Tunisia (TUN). The scale bar indicates the number of differing alleles and is equivalent to 100 allele differences. (For further details, see Appendix A and Table S3).

**Figure 4:**
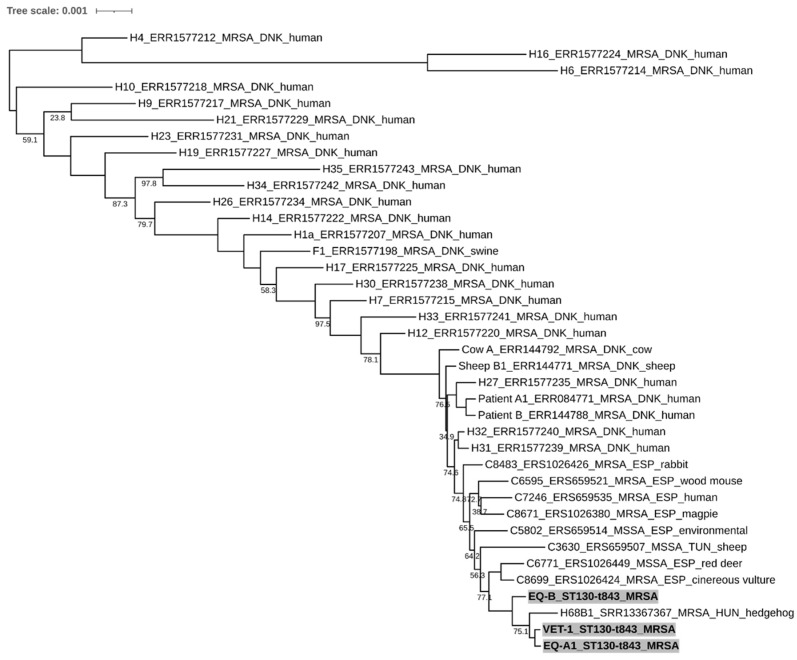
Neighbour-joining tree from the SNP-based phylogenetic analysis. The phylogenetic relationship between the two equine (EQ-A1, EQ-B) and the human (VET-1) originated *mecC*-positive isolates from Hungary (highlighted with grey background) in comparison of other selected 32 *mecC*-positive MRSA and three MSSA CC130 isolates from Europe (Denmark (DNK), Hungary (HUN), Spain (ESP), and Tunisia (TUN)). The scale bar indicates number and type of substitutions. Only bootstrap values lower than 100 are shown. (For further details, see Appendix A and Table S3).

All whole genome sequenced isolates showed MLST sequence type (ST) 130 and *spa* type t843, with the latter in agreement with the Sanger sequencing-based *spa* typing. EQ-A1 and VET-1 show the closest relationship by the core genome MLST analysis, differing by only five alleles. Of note, these isolates clustered together with the Hungarian hedgehog *mecC*-MRSA isolate (H68B1), showing 128 and 127 allelic differences, but were markedly separated from the EQ-B strain by 232 and 231 allelic differences, respectively. The close relationship between the EQ-A1 and the VET-1 was reinforced by the SNP analysis, which showed 729 substitutions, while they differed by 2522–2598 (mean: 2563) SNPs from both the hedgehog and the EQ-B isolates. Interestingly, the SNP difference between EQ-B and the hedgehog isolate was 4051 substitutions (Figure 3 and Figure 4) (Table S2).

Except for the *mecC* gene and beta lactamase enzyme gene (*blaZ*), no other resistance determinants were identified in the whole genome sequenced isolates, which is in good agreement with the phenotypic data. All of these isolates harboured the *SCCmec* XI element and carried the genes encoding for two pore-forming virulence factors: gamma-hemolysin genes *hlgA*, *hlgB* and *hlgC*, and the leukocidin gene *lukED*. The epidermal cell differentiation inhibitor B gene (*edinB*) and the exfoliative toxin E (*etE*, previously *etD2*), in close proximity to each other, were also identified. The human-specific immune evasion cluster (IEC) and *lukS-PV/lukF-PV* gene were missing from all three sequenced MRSA strains. 

## 3. Discussion

In horses, MRSA infection can result in asymptomatic carriage or in various clinical outcomes, the latter ranging from mild, benign skin and soft tissue infections to severe necrotic pneumonia and fatal sepsis. Animals with an underlying disease are more prone to develop clinical infection, but an iatrogenic contamination of tissues is also suspected as an important risk factor [4].

In this study, the EQ-A horse had COPD and a MRSA thrombophlebitis linked to intravenous cannulation, while the EQ-B horse and the veterinarian were only asymptomatic carriers.

EQ-A1, EQ-B, and VET-1 samples shared the same genotype, all belonged to ST130-t843. This ST130 sequence type normally lacks the human host adaption-associated immune evasion cluster—just like the EQ-A1, EQ-B, VET-1 isolates in this study—therefore, in these cases, animal origins can be suspected [31,34].

The WGS-based cgMLST and SNP analyses partly resolved the apparent genetic homogeneity of the strains (Figure 3 and Figure 4). The closest relationship could be found between the EQ-A1 and the VET-1 isolates, characterized by only five allelic differences in the cgMLST analysis (Table S2). This value is in the range of the estimated annual allelic variation of *S. aureus* clonal lineages within a host, according to a recent study [35]. Results of the SNP-based analysis were in line with those obtained by the cgMLST approach. However, our isolates showed more genetic distances than expected. This is not surprising, since we investigated the SNP content of the complete genome, rather than using only the core genome data. As a consequence, the thresholds suggested for close relatedness, ≤2–5.8 and ≤15 core genome SNP [35,36], could not be applied. Nevertheless, the apparent differences between the cgMLST and SNP-based trees can also be explained by the differences between the whole genome and core genome content. The findings further support the hypothesis that a horse-to-human or human-to-horse MRSA transmission has happened in the case of the EQ-A1 and VET-1 isolates, and only small numbers of mutations emerged during the adaptation to the new host species. Furthermore, isolates EQ-A1 and VET-1 showed high similarity with the EQ-B and the Hungarian hedgehog isolate as well. Moreover, these Hungarian isolates are separated from the other European and Tunisian *mecC*-MRSA samples, according to the SNP phylogenetic tree. These results suggest that *mecC*-MRSA strains have a rather defined geographical distribution, and the isolates examined in this manuscript may have derived from the cluster circulating in Hungary.

Transmission of *mecC*-MRSA between animals (cows and sheep) and humans has already beenconfirmed in two human infections by whole genome sequencing. Patient 1 had a blood stream infection, while Patient 2 was treated with a wound infection caused by *mecC*-MRSA. In both cases the screened household animals were only asymptomatic carriers of the pathogen. Phylogenetic analysis of the core genome using SNPs revealed that the isolates recovered from the same farm, from humans and animal, were closely related [37]. In a Danish study, swine *mecC*-MRSA isolates were compared to human MRSA strains and a close affiliation was found between the swine samples and human isolates from the same farm municipality, indicating transmission between humans and swine [38]. Furthermore the WGS data of these Danish isolates were used in our cgMLST and SNP comparison (Figure 3 and Figure 4) 

According to the previous data, *mecC* carrying MRSA is sporadic in horses, and strains with different genetic backgrounds are involved. In 2014, the first infection with *mecC*-MRSA CC130-t843 was isolated from a foot wound of a horse in France [18]. In the next year, another infection was described in Germany with the same sequence type [12]. The very first *mecC*-MRSA isolates from horses were also documented in France, but these had different sequence types: in 2012, a (CC130) ST1245-t6220 MRSA was isolated from a respiratory tract infection; in 2013, (CC49) ST49-t208 was cultured from a skin infection; and in 2013, a ST130-t11015 was found in a pododermatitis case [18].

In a previous study in Hungary between 2011–2016, MRSA was detected from a sequence of nosocomial outbreaks in the university equine clinic [14]. In this study, the equine clinic-associated subclone of ST398-t011 was the exclusive MRSA type. Based on these former observations, the presence of the ST130-t843 (*mecC*+) isolates in the Hungarian horse population and in the equine health care setting is an unusual and novel finding [14]. Until now, only one *mecC*-MRSA isolate has been reported from Hungary, isolated from a hedgehog, which belonged to (CC130) ST6736-t19701, and this type of resistance was expected to appear in new environments such as the veterinary and human health care systems. 

In the abovementioned study [14], not only horses but also veterinary personnel of the equine clinic were screened. Most of the human isolates shared the MRSA genotype of the horses, proving that humans can be colonized with these bacteria and can be a potential risk in human-to-horse and horse-to-human transmission and infection [14]. This information further highlights the importance of the current Hungarian ST130-t843 isolates as the same clone was isolated from a veterinarian, who was in direct contact with a confirmed infected horse. This is the first description of direct transmission of a *mecC*-carrying MRSA between a horse and its veterinarian. 

Since neither the source nor the direction of the MRSA transmission between the veterinarian and its patient could be determined with certainty, we can only talk about long-term colonization with caution in the case of the veterinarian. Even so, the possibility of *mecC*-MRSA transmission must raise the attention of equine professionals to biosecurity when treating risk patients in order to avoid human infection or colonization by MRSA. 

Detection of *mecC*-MRSA isolates may pose difficulties, as these bacteria may show a different phenotypical appearance and carry a different *mec* gene compared to the more common *mecA*-MRSA isolates. Because of the differences, there is a risk that these isolates would be missed and not identified as MRSA during the routine laboratory diagnostics. 

Based on the growth characteristics of the *mecC*-MRSA isolates on Columbia blood agar and on selective chromogenic media (slow colony formation, small colony size) in this study, these may be considered small colony variant (SCV) S. aureus isolates. SCV *S. aureus* has been reported to cause difficulties in the identification and in the in vivo antimicrobial therapy because of their slow growth, slow rate of cell division, and their reduced metabolic activity [39,40].

If MRSA screening is performed with methods identifying the *mecA* gene (e.g., PCR test), or the protein product of the gene (PBP2a), *mecC*-MRSA strains will always be overlooked [41]. Another difference is that the protein product of the *mecC*-gene (PBP2c) has a different optimum temperature compared to the classical PBP2a, and at higher temperatures, it collapses, e.g., at 37 °C, it has a lower activity. The two PBPs also have a different affinity for cefoxitin and oxacillin [41]. The PBP2c is frequently described as cefoxitin-resistant, but oxacillin-susceptible, and only the antibiotic susceptibility test with a cefoxitin disk is able to categorize *mecC*-MRSA properly [42]. Therefore cefoxitin was confirmed as a superior marker to detect MRSA compared to oxacillin [42]. According to the current EUCAST guidelines, cefoxitin disk tests are recommended to screen for MRSA isolates [43]. 

All *mecC*-MRSA strains examined in this study were sensitive to the combination of amoxicillin and the ß-lactam inhibitor clavulanic acid in vitro. The biological differences demonstrated between *mecA*- and *mecC*-encoded PBP2a and PBP2c were discussed above [41,44]. Furthermore, it is suspected that in the *mecC*-MRSA strains, the broad-spectrum ß-lactam resistance is mediated by not only the *mecC* gene but the combination of both PBP2c and the distinct ß-lactamase encoded by the *blaZ*, which is part of *mecC*-encoding *SCCmec* type XI cassette [44]. Thus, the biological differences between PBP2a and PBP2c have the potential to be used as a novel approach for the treatment of *mecC*-MRSA infections [44].

## 4. Materials and Methods

### 4.1. Sample Processing, Isolation, and Identification of MRSA

Isolates VET-1, VET-2 and EQ-B were cultured using a pre-enrichment method. Swabs were placed in 5 mL Mueller-Hinton broth supplemented with 6.5% NaCl, and incubated at 37 °C for 16–24 h. A loopful (~10 µL) of the pre-enrichment medium was spread on chromogenic agar plates selective for *S. aureus* and MRSA, respectively (BD BBL CHROMagar Staph aureus and BD BBL CHROMagar MRSA II, Diagon Ltd., Hungary), and incubated at 37 °C for 24–48 h. Bacterial growth controls (ATCC 25923 for *S. aureus* and ATCC 43300 for MRSA-selective media) were included in each step during the protocol to monitor the performance of the different media. Isolates EQ-A1 and EQ-A2 were primarily cultured on Columbia blood agar, and the methicillin resistance was confirmed by first-line cefoxitin disc-diffusion testing, as recommended by the EUCAST standards [43]. Multiplex PCR was used for the detection of the *spa* gene, a species specific marker of *S. aureus*, *mecA* and *mecC* genes, confirming methicillin resistance and *lukS-PV/lukF-PV* as the marker of a human-related virulence factor Panton-Valentine leukocidin [45]. All strains were stored at −80 °C until further investigations.

In accordance with the Act XXVIII of 1998 and Government Decree 40/2013 (II. 14), our research is not defined as an animal study, and thus, it does not require further legal or ethical permission. The veterinarian has participated voluntarily in the survey and provided an informed written consent. The human sampling protocol was supervised and approved by the Committee of Science and Research Ethics, Medical Research Council, Ministry of Human Capacities, Hungary, as a protocol that does not require medical intervention (No. 42323-2/2019/EKU).

### 4.2. Antibiotic Susceptibility Testing

Minimal inhibitory concentrations of 15 antibiotic compounds were determined by using a custom-made panel (Micronaut-S R56, Merlin Diagnostika, Berlin, Germany). The disc diffusion method was also used to test the antibiotic susceptibility of strains to 11 addditional antimicrobial agents (Biolab, Budapest, Hungary). Breakpoints were evaluated according to the EUCAST [43], where they were available, otherwise CLSI standards were applied [46,47]. In the case of streptomycin, where there is no breakpoint reported, resistance was further investigated for the presence of a resistance mechanism by testing isolates for the presence of *aadE* and *str* genes [48,49]. Both genes are known to confer resistance to streptomycin in staphylococci [50]. Antibiotics were used as indicators of possible resistance mechanisms and not for clinical purposes. The compounds tested, along with their MIC ranges, breakpoints, and results are summarised in Table S1.

### 4.3. Genotyping and Whole-Genome Sequencing of MRSA Strains

Initial genotyping of all staphylococcus isolates was performed using *spa* typing, classical multilocus-sequence typing methods and a set of multiplex PCRs to determine the Staphylococcus cassette chromosome *mec* (SSC*mec*) type, as recently described [51]. To assess the genetic relatedness of the three equine and two human *mecC*-positive isolates, a standard PFGE method was used [52,53]. The *mecC*-MRSA isolates were digested with the *SmaI* restriction endonuclease enzyme. The cluster analysis of the PFGE gel pictures was performed by the Fingerprinting II software (Bio-Rad, Marnes-la-Coquette, France).

Based on the PFGE results, one isolate from each horse (isolate EQ-B and EQ-A1) and the first human isolate (VET-1) were chosen for whole-genome sequencing in the sequencing facility of BIOMI Ltd., according to a previously described method on an Illumina MiSeq platfom [54]. Sequence data management was carried out by using an in-house pipeline. This included the de novo assembly of genomes and the analysis of genetic relatedness based on core genome multilocus sequence typing (cgMLST) and single nucleotide polymorphisms (SNPs). For further details, please see Appendix A. MLST and *spa* type of assembled draft genomes was assessed using the MLST v2.0 and spaTpyer v1.0 [55,56]. To screen for genetic markers associated with antimicrobial resistance and virulence, we used ResFinder v4.0 and VirulenceFinder v2.0 with default settings, respectively [57,58]. The SCC*mec* type was identified by using the SCC*mec*Finder v1.2 [59]. The genetic vicinity of the identified virulence genes was visualized and further investigated by using Geneious Prime version 2022.1.1. (Biomatters Ltd., Auckland, New Zealand). All nucleotide sequences are available in the Sequence Read Archive under the BioProject PRJNA876088.

We also selected whole genome sequence data from previous studies investigating methicillin-resistant or -sensitive CC130 strains isolated in Europe and Tunisia for comparative purposes (Table S3). The selected strains originated from humans and various animal hosts, and some from possible cases of zoonotic transmission events [31,37,38]. A recently described hedgehog-originated *mecC*-carrying MRSA strain from Hungary was also involved in the analysis [60]. Sequence data were managed as previously described and included only in the cgMLST and SNP-based analyses. 

## 5. Conclusions

The emergence of the *mecC* gene in Hungary draws attention to the importance of the appropriate diagnostic techniques when it comes to MRSA detection in microbiological laboratories. Automated systems may fail to detect the MRSA isolate containing only the *mecC* gene. The same is true for identification protocols where only the presence of the *mecA* gene, or PBP2a, is examined. Therefore, the implementation of the most current and revised antibiotic susceptibility recommendations is important, and molecular assays should be used to verify the presence of the *mecC* gene whenever possible. 

Based on the WGS analysis of the *mecC*-MRSA isolates, direct bacterial transmission can be suspected between a horse and a human, as the *mecC*-MRSA carrying veterinarian was in direct contact with the *mecC*-MRSA infected horse. Furthermore, only minimal differences were detectable with the cgMLST and SNP analyses between the two concerned isolates, although the direction of the MRSA transmission could not be determined with certainty. The isolates lacked the human-associated IEC and *lukS-PV/lukF-PV* gene, which suggests that the ST130-t843 *mecC*-MRSA originally developed in animal hosts. This idea is further supported by the fact that we previously detected *mecC*-MRSA in hedgehogs, but none has been documented in humans in Hungary so far. Due to the possibility of human-to-animal-to-human transmission and the long-term colonization in the veterinarian in this case, biosecurity should also be a priority in equine practices outside the clinical environment, with special attention to personal hygiene when treating patients at risk of MRSA infection.

## Data Availability

The data presented in this study are available in Supplementary materials. All nucleotide sequences obtained during this study are available in the Sequence Read Archive under the BioProject PRJNA876088.

## Supplementary Materials

The following supporting information can be downloaded at: https://www.mdpi.com/article/10.3390/antibiotics12020408/s1, Figure S1: Raw images: the original *S. aureus* PFGE gel pictures; Table S1: Antibiotic susceptibility tests of the *mecC*-MRSA isolates; Table S2. MLST allele difference and SNP difference matrix in the analysed CC130 strains. Table S3. List of the selected CC130 strains used for comparative purposes.

## Appendix A

Initial quality of the raw sequencing data was assessed with FastQC v0.11.7 [61]. Trimmomatic v0.39 [62] was used for quality trimming of the sequences with the following settings: LEADING:3, TRAILING:3, SLIDINGWINDOW:4:30, MINLEN:50. Quality trimmed reads were assembled into contigs with MEGAHIT v1.2.9 [63]. cgMLST analysis was performed on the contigs with chewBBACA v.2.8.5 [64], using the cgMLST scheme of *S. aureus*, available at the cgmlst.org website [65]. Neighbour-joining trees were constructed from the cgMLST profiles with the ape v5.6.2 R package using the allele difference matrix of 1861 core genome loci [66,67]. For SNP variant call analysis, reads were aligned to the *S. aureus* reference sequence NC_007795.1 (downloaded at: 28 September 2021) with Bowtie2 v2.4.2 [68]. To retain the most aligned reads for further analysis, paired and unpaired bam files were merged together and duplicates were removed with Picard v2.21.4 [69]. Samtools v1.10 [70,71] was used for pileup construction and SNPs were called with VarScan v2.4.4 [72], using the mpileup2snp command with the following parameters: --min-reads2 4, --min-coverage 30, --min-avg-qual 20, --min-var-freq 0.90, --strand-filter 1, --output-vcf 1. Consensus genomes were constructed with bcftools v1.9 [70] and masked at positions where the depth of coverage was less than 30. Maximum likelihood phylogenetic analysis of the consensus sequences was performed in R environment [67] with the aid of the ape v5.6.2 [66], phangorn v2.9.0 [73] and Biostrings v2.60.2 [74] R packages. GTR + G + I was selected as the optimal nucleotide substitution model with the modelTest function of the phangorn package. Reliability of tree branches was tested with a bootstrap analysis with 1000 iterations. All phylogenetic trees were visualised in iTOL [75].
