# Supplementary material for: Genomic Evidence for Direct Transmission of mecC-MRSA between a Horse and Its Veterinarian
Source: Antibiotics (Basel). 2023 Feb 17;12(2):408. doi: 10.3390/antibiotics12020408 (PMC9952710; doi:10.3390/antibiotics12020408)
Supplement: Supplementary file 1 [file antibiotics-12-00408-s001.zip › Figure S1 - new.pdf]

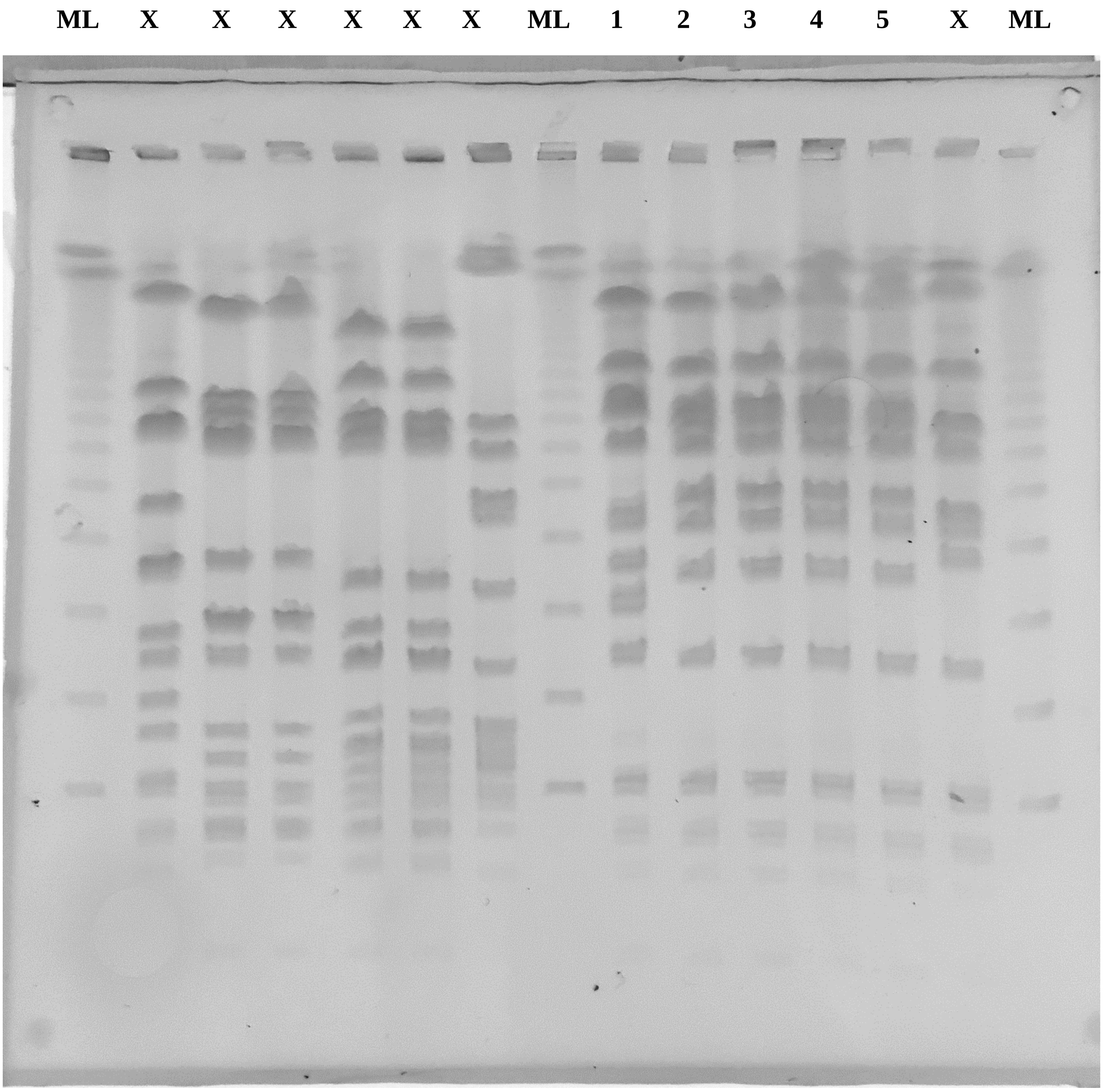

Figure S1: Raw images: the original *S. aureus* PFGE gel pictures.

ML: Lambda (λ) DNA Marker (CHEF DNA size standard; catalog no. 170-3635; Bio-Rad). X: strains are not used in this study. 1: EQ-B. 2: EQ-A1. 3: EQ-A2. 4: VET1. 5: VET2.

Fig 1 was generated from these original PFGE pictures.
